# Supplementary figures and images for: A novel chimeric RNA originating from BmCPV S4 and Bombyx mori HDAC11 transcripts regulates virus proliferation
Source: PLoS Pathog. 2023 Dec 4;19(12):e1011184. doi: 10.1371/journal.ppat.1011184 (PMC10721177; doi:10.1371/journal.ppat.1011184)

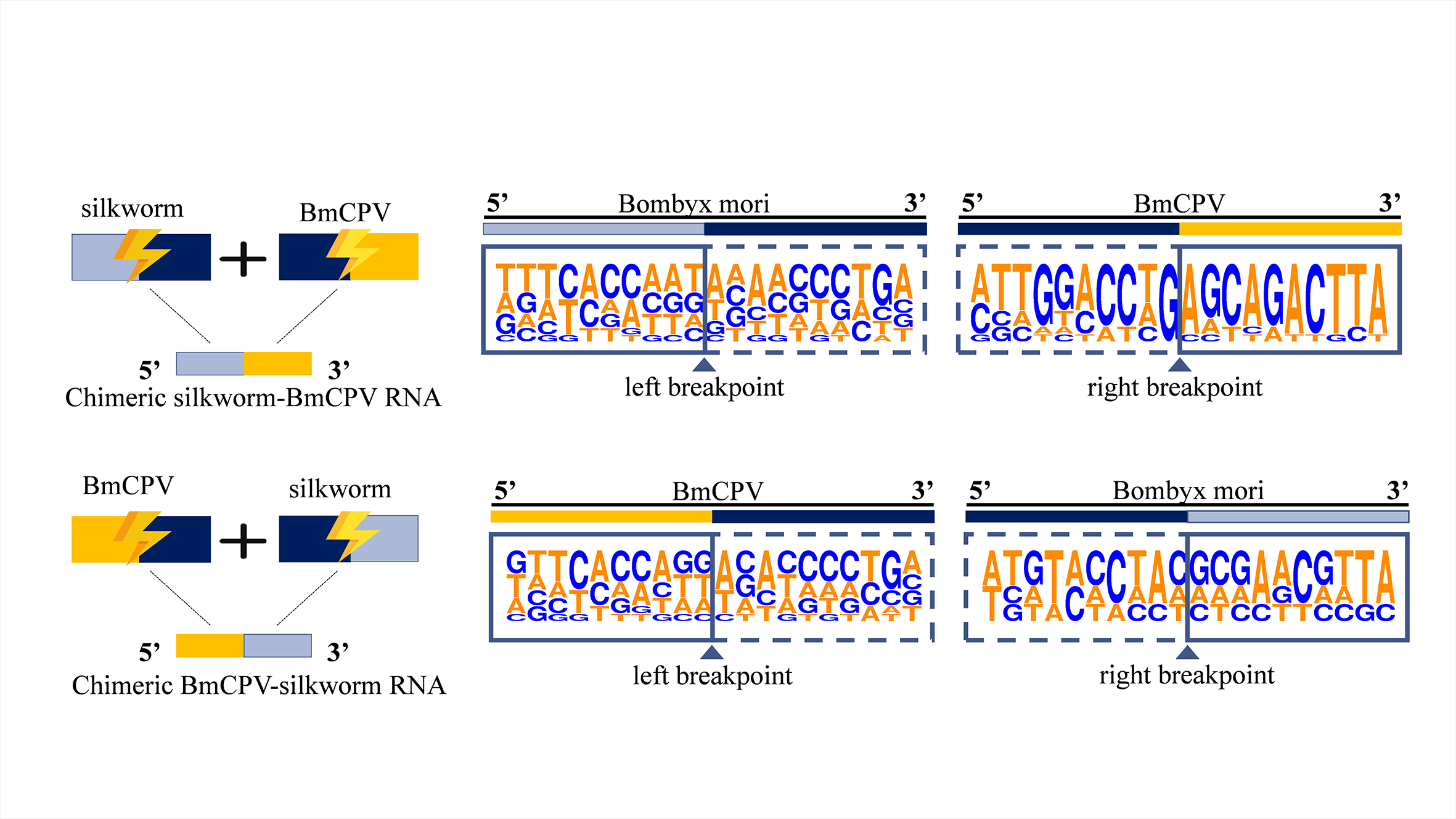

Supplement: S1 Fig — (TIF) [file ppat.1011184.s001.tif]

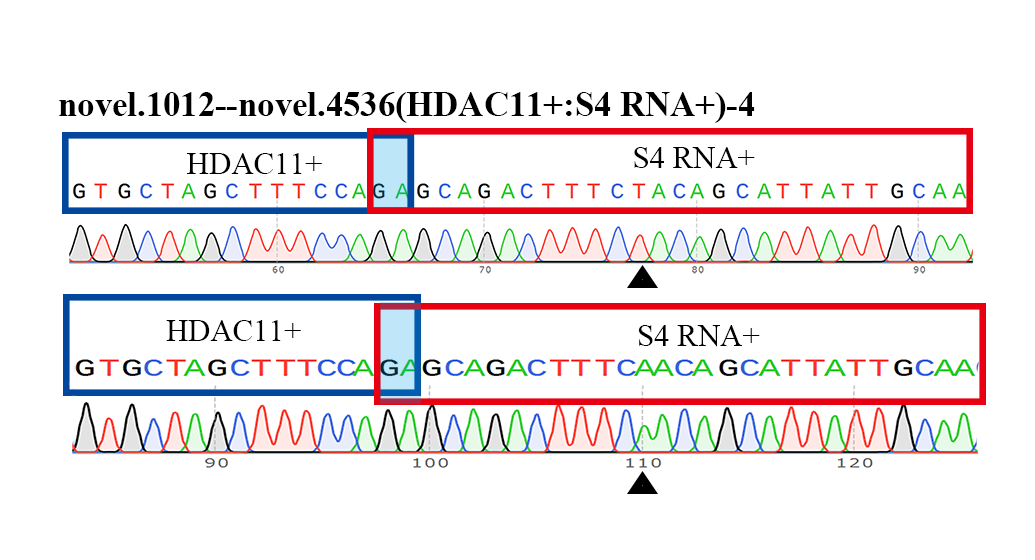

Supplement: S2 Fig — (TIF) [file ppat.1011184.s002.tif]
