# Supplementary material for: A novel chimeric RNA originating from BmCPV S4 and Bombyx mori HDAC11 transcripts regulates virus proliferation
Source: PLoS Pathog. 2023 Dec 4;19(12):e1011184. doi: 10.1371/journal.ppat.1011184 (PMC10721177; doi:10.1371/journal.ppat.1011184)
Supplement: S1 Table — (PDF) [file ppat.1011184.s004.pdf]

FusionName	Junction ReadCoun	Spanning FragCoun	SpliceType	LeftGene	LeftBreakpoint	RightGene	RightBreakpoint	FFPM	LeftBreakDin	LeftBreakEntropy	RightB	RightBreakEntrop	annots
novel.2201--novel.4520	143	0	INCL_NON_REF_SPLICE	novel.2201	BMSK_chr20:2995833:-	novel.4520	GenBank_GQ150538.1_S7:1241:+	3.6101	TT	1.5219	AG	1.7465	["INTERCHROMOSOMAL[BMSK_chr20--GenBank_GQ150538.1_S7]"]
novel.4521--novel.2102	58	26	INCL_NON_REF_SPLICE	novel.4521	GenBank_GQ150538.1_S7:1243:-	novel.2102	BMSK_chr2:7356934:-	2.1206	TT	1.8256	TC	1.4256	["INTERCHROMOSOMAL[GenBank_GQ150538.1_S7--BMSK_chr2]"]
novel.4533--novel.474	47	7	INCL_NON_REF_SPLICE	novel.4533	GenBank_GQ924588.1_circ_000018_S9:333:-	novel.474	BMSK_chr11:4416195:-	1.187	CG	1.9656	AC	1.8062	["INTERCHROMOSOMAL[GenBank_GQ924588.1_circ_000018_S9--BMSK_chr11]"]
novel.4531--novel.474	40	22	INCL_NON_REF_SPLICE	novel.4531	GenBank_GQ924587.1_circ_000165_S3:399:-	novel.474	BMSK_chr11:4416195:-	1.3628	CT	1.8892	AC	1.8062	["INTERCHROMOSOMAL[GenBank_GQ924587.1_circ_000165_S3--BMSK_chr11]"]
novel.4533--novel.474	33	4	INCL_NON_REF_SPLICE	novel.4533	GenBank_GQ924588.1_circ_000018_S9:333:-	novel.474	BMSK_chr11:4416195:-	0.9278	CG	1.9656	AC	1.8062	["INTERCHROMOSOMAL[GenBank_GQ924588.1_circ_000018_S9--BMSK_chr11]"]
novel.4533--novel.474	23	1	INCL_NON_REF_SPLICE	novel.4533	GenBank_GQ924588.1_circ_000018_S9:333:-	novel.474	BMSK_chr11:4416195:-	0.6058	CG	1.9656	AC	1.8062	["INTERCHROMOSOMAL[GenBank_GQ924588.1_circ_000018_S9--BMSK_chr11]"]
novel.4525--novel.474	18	20	INCL_NON_REF_SPLICE	novel.4525	GenBank_GQ294468.1_S5:164:-	novel.474	BMSK_chr11:4416195:-	0.8352	CT	1.8323	AC	1.8062	["INTERCHROMOSOMAL[GenBank_GQ294468.1_S5--BMSK_chr11]"]
novel.4531--novel.474	14	10	INCL_NON_REF_SPLICE	novel.4531	GenBank_GQ924587.1_circ_000165_S3:399:-	novel.474	BMSK_chr11:4416195:-	0.6017	CT	1.8892	AC	1.8062	["INTERCHROMOSOMAL[GenBank_GQ924587.1_circ_000165_S3--BMSK_chr11]"]
novel.4525--novel.474	12	7	INCL_NON_REF_SPLICE	novel.4525	GenBank_GQ294468.1_S5:164:-	novel.474	BMSK_chr11:4416195:-	0.4764	CT	1.8323	AC	1.8062	["INTERCHROMOSOMAL[GenBank_GQ294468.1_S5--BMSK_chr11]"]
novel.4537--novel.474	12	24	INCL_NON_REF_SPLICE	novel.4537	GenBank_GU323606.1_circ_000382_S4:884:-	novel.474	BMSK_chr11:4416195:-	0.7913	CA	1.9329	AC	1.8062	["INTERCHROMOSOMAL[GenBank_GU323606.1_circ_000382_S4--BMSK_chr11]"]
novel.4523--novel.474	11	8	INCL_NON_REF_SPLICE	novel.4523	GenBank_GQ150539.1_S8:251:-	novel.474	BMSK_chr11:4416195:-	0.4176	CG	1.8892	AC	1.8062	["INTERCHROMOSOMAL[GenBank_GQ150539.1_S8--BMSK_chr11]"]
novel.4523--novel.474	11	4	INCL_NON_REF_SPLICE	novel.4523	GenBank_GQ150539.1_S8:251:-	novel.474	BMSK_chr11:4416195:-	0.3761	CG	1.8892	AC	1.8062	["INTERCHROMOSOMAL[GenBank_GQ150539.1_S8--BMSK_chr11]"]
novel.4527--novel.474	10	6	INCL_NON_REF_SPLICE	novel.4527	GenBank_GQ294469.1_S6:368:-	novel.474	BMSK_chr11:4416195:-	0.3517	CT	1.8295	AC	1.8062	["INTERCHROMOSOMAL[GenBank_GQ294469.1_S6--BMSK_chr11]"]
novel.4531--novel.474	9	9	INCL_NON_REF_SPLICE	novel.4531	GenBank_GQ924587.1_circ_000165_S3:399:-	novel.474	BMSK_chr11:4416195:-	0.4544	CT	1.8892	AC	1.8062	["INTERCHROMOSOMAL[GenBank_GQ924587.1_circ_000165_S3--BMSK_chr11]"]
novel.4535--novel.474	9	18	INCL_NON_REF_SPLICE	novel.4535	GenBank_GU323605.1_S1:390:-	novel.474	BMSK_chr11:4416195:-	0.5934	CT	1.7968	AC	1.8062	["INTERCHROMOSOMAL[GenBank_GU323605.1_S1--BMSK_chr11]"]
novel.4537--novel.474	9	6	INCL_NON_REF_SPLICE	novel.4537	GenBank_GU323606.1_circ_000382_S4:884:-	novel.474	BMSK_chr11:4416195:-	0.3787	CA	1.9329	AC	1.8062	["INTERCHROMOSOMAL[GenBank_GU323606.1_circ_000382_S4--BMSK_chr11]"]
novel.4525--novel.474	8	10	INCL_NON_REF_SPLICE	novel.4525	GenBank_GQ294468.1_S5:164:-	novel.474	BMSK_chr11:4416195:-	0.4545	CT	1.8323	AC	1.8062	["INTERCHROMOSOMAL[GenBank_GQ294468.1_S5--BMSK_chr11]"]
novel.4537--novel.2102	7	1	INCL_NON_REF_SPLICE	novel.4537	GenBank_GU323606.1_circ_000382_S4:885:-	novel.2102	BMSK_chr2:7356933:-	0.2006	TC	1.9899	CT	1.5546	["INTERCHROMOSOMAL[GenBank_GU323606.1_circ_000382_S4--BMSK_chr2]"]
novel.4537--novel.474	7	10	INCL_NON_REF_SPLICE	novel.4537	GenBank_GU323606.1_circ_000382_S4:884:-	novel.474	BMSK_chr11:4416195:-	0.4262	CA	1.9329	AC	1.8062	["INTERCHROMOSOMAL[GenBank_GU323606.1_circ_000382_S4--BMSK_chr11]"]
novel.4529--novel.474	6	7	INCL_NON_REF_SPLICE	novel.4529	GenBank_GQ924586.1_S2:401:-	novel.474	BMSK_chr11:4416195:-	0.3259	CT	1.9899	AC	1.8062	["INTERCHROMOSOMAL[GenBank_GQ924586.1_S2--BMSK_chr11]"]
novel.4525--novel.2102	5	2	INCL_NON_REF_SPLICE	novel.4525	GenBank_GQ294468.1_S5:165:-	novel.2102	BMSK_chr2:7356933:-	0.1539	TC	1.8323	CT	1.5546	["INTERCHROMOSOMAL[GenBank_GQ294468.1_S5--BMSK_chr2]"]
novel.4527--novel.474	5	6	INCL_NON_REF_SPLICE	novel.4527	GenBank_GQ294469.1_S6:368:-	novel.474	BMSK_chr11:4416195:-	0.2758	CT	1.8295	AC	1.8062	["INTERCHROMOSOMAL[GenBank_GQ294469.1_S6--BMSK_chr11]"]
novel.2201--novel.4532	4	0	INCL_NON_REF_SPLICE	novel.2201	BMSK_chr20:2995833:-	novel.4532	GenBank_GQ924588.1_circ_000018_S9:333:+	0.101	TT	1.5219	CG	1.9656	["INTERCHROMOSOMAL[BMSK_chr20--GenBank_GQ924588.1_circ_000018_S9]"]
novel.4523--novel.474	3	5	INCL_NON_REF_SPLICE	novel.4523	GenBank_GQ150539.1_S8:251:-	novel.474	BMSK_chr11:4416195:-	0.2019	CG	1.8892	AC	1.8062	["INTERCHROMOSOMAL[GenBank_GQ150539.1_S8--BMSK_chr11]"]
novel.4525--novel.2102	3	2	INCL_NON_REF_SPLICE	novel.4525	GenBank_GQ294468.1_S5:165:-	novel.2102	BMSK_chr2:7356933:-	0.1253	TC	1.8323	CT	1.5546	["INTERCHROMOSOMAL[GenBank_GQ294468.1_S5--BMSK_chr2]"]
novel.4527--novel.2102	3	1	INCL_NON_REF_SPLICE	novel.4527	GenBank_GQ294469.1_S6:369:-	novel.2102	BMSK_chr2:7356925:-	0.1003	TC	1.8295	AG	1.7232	["INTERCHROMOSOMAL[GenBank_GQ294469.1_S6--BMSK_chr2]"]
novel.4529--novel.474	3	21	INCL_NON_REF_SPLICE	novel.4529	GenBank_GQ924586.1_S2:401:-	novel.474	BMSK_chr11:4416197:-	0.5275	CT	1.9899	CG	1.7819	["INTERCHROMOSOMAL[GenBank_GQ924586.1_S2--BMSK_chr11]"]
novel.4531--novel.1087	3	2	INCL_NON_REF_SPLICE	novel.4531	GenBank_GQ924587.1_circ_000165_S3:399:-	novel.1087	BMSK_chr14:9928602:-	0.1099	CT	1.8892	TC	1.9329	["INTERCHROMOSOMAL[GenBank_GQ924587.1_circ_000165_S3--BMSK_chr14]"]
novel.4533--novel.1088	3	3	INCL_NON_REF_SPLICE	novel.4533	GenBank_GQ924588.1_circ_000018_S9:334:-	novel.1088	BMSK_chr14:9932346:-	0.1514	TC	1.9899	GA	1.8892	["INTERCHROMOSOMAL[GenBank_GQ924588.1_circ_000018_S9--BMSK_chr14]"]
novel.4535--novel.474	3	6	INCL_NON_REF_SPLICE	novel.4535	GenBank_GU323605.1_S1:390:-	novel.474	BMSK_chr11:4416195:-	0.2256	CT	1.7968	AC	1.8062	["INTERCHROMOSOMAL[GenBank_GU323605.1_S1--BMSK_chr11]"]
novel.1012--novel.4530	3	2	INCL_NON_REF_SPLICE	novel.1012	BMSK_chr14:9928600:+	novel.4530	GenBank_GQ924587.1_circ_000165_S3:399:+	0.1099	GT	1.9329	AG	1.8892	["INTERCHROMOSOMAL[BMSK_chr14--GenBank_GQ924587.1_circ_000165_S3]"]
novel.1012--novel.4532	3	2	INCL_NON_REF_SPLICE	novel.1012	BMSK_chr14:9928752:+	novel.4532	GenBank_GQ924588.1_circ_000018_S9:333:+	0.1099	TC	1.9086	CG	1.9656	["INTERCHROMOSOMAL[BMSK_chr14--GenBank_GQ924588.1_circ_000018_S9]"]
novel.1012--novel.4536	3	2	INCL_NON_REF_SPLICE	novel.1012	BMSK_chr14:9928754:+	novel.4536	GenBank_GU323606.1_circ_000382_S4:884:+	0.1099	GA	1.9329	TG	1.9329	["INTERCHROMOSOMAL[BMSK_chr14--GenBank_GU323606.1_circ_000382_S4]"]
novel.1013--novel.4532	3	3	INCL_NON_REF_SPLICE	novel.1013	BMSK_chr14:9932346:+	novel.4532	GenBank_GQ924588.1_circ_000018_S9:334:+	0.1514	TC	1.8892	GA	1.9899	["INTERCHROMOSOMAL[BMSK_chr14--GenBank_GQ924588.1_circ_000018_S9]"]
