# Supplementary material for: A novel chimeric RNA originating from BmCPV S4 and Bombyx mori HDAC11 transcripts regulates virus proliferation
Source: PLoS Pathog. 2023 Dec 4;19(12):e1011184. doi: 10.1371/journal.ppat.1011184 (PMC10721177; doi:10.1371/journal.ppat.1011184)
Supplement: S2 Table — (PDF) [file ppat.1011184.s005.pdf]

Chimeric RNA/gene	Forward Primer and Reverse Primer	Sequence
(S3-:LsrRNA-)4	(S3-:LsrRNA-)4-F	ACTGCACGTTCCATTGTTGC
	(S3-:LsrRNA-)4-R	CCTGAGCGTTCGAGTTCCAT
(S4-:LsrRNA-)6	(S4-:LsrRNA-)6-F	TTCTGGCAATGGCGCTATCA
	(S4-:LsrRNA-)6-R	CCTGAGCGTTCGAGTTCCAT
(S7-:fcaL43P13 RNA+)2	(S7-:fcaL43P13 RNA+)2-F	GCAGATCTTGTCACTCCCCC
	(S7-:fcaL43P13 RNA+)2-R	TTCCCTGCTACCCGCTCTAT
(U6RNA+:S7+)1	(U6RNA+:S7+)1-F	TGACAAGGATGGAACACACAA
	(U6RNA+:S7+)1-R	AGCGCAGATCTTGTCACTCC
(U6RNA+:S9+)13	(U6RNA+:S9+)13-F	AACACACAATTTGGTTAAGGCACT
	(U6RNA+:S9+)13-R	TTGGCACGAAAAATGGAGCG
(Bm_160RNA+:S9+)21	(Bm_160RNA+:S9+)21-F	GTCCGTTTCTACACAGGAAAG
	(Bm_160RNA+:S9+)21-R	GGCTAACGACCCGAGTGCCCT
(HDAC11+:S4 RNA+)20-123	(HDAC11+:S4 RNA+)20-123-F	CCGGTTCAGCCTTGACCTTA
	(HDAC11+:S4 RNA+)20-123-R	ACGTCTGAGTTGATCGTTCGC
(HDAC11+:S4 RNA+)20-456	(HDAC11+:S4 RNA+)20-456-F	CCAGAGTCAAGTCAGCAGCA
	(HDAC11+:S4 RNA+)20-456-R	GCACAAATAACTGCCGATCCAA
S9 RNA+:S7 RNA+	S9 RNA+:S7 RNA+ F	AATCCCAGGCGTAAACCGAA
	S9 RNA+:S7 RNA+ R	CAGACGCGCTGACGTATTTG
S7 RNA-:S5 RNA -	S7 RNA-:S5 RNA - F	GCAGCGCAGATCTTGTCACTCC
	S7 RNA-:S5 RNA - R	TTGCTCGTTAAGGGCGACTT
vp1	CPV-S1F	GGTCTCGACGTGAATACCGA
	CPV-S1R	TCGTCTGCTTCACTAGCACG
TIF-4A	TIF4A-F	GAATGGACCCTGGGACACTT
	TIF4A-R	CTGACTGGGCTTGAGCGATA
(HDAC11+:S4 RNA+)20	adapterHCPV-20-4-F	GTGCTAGCTTTCCAGAGCAGAC
	adapterHCPV-20-4-R	GTTGCTCCAGTTACAGTCAGCG
(HDAC11+:S4 RNA+)20	qHCPV20-4-F	GAGTCAAGTCAGCAGCAACG
	qHCPV20-4-R	CATGCGTCTGTATAAACG
GFP	T7GFP-F	TAATACGACTCACTATAGGGTCTGTCAG TGGAGAGGGTGAA
	GFP-R	CCCAGCAGCAGTTACAAACTC
T7HDAC11	T7HDAC11-F	TAATACGACTCACTATAGGGAATGGGAA GTCCGTTTCTACACAGG
	HDAC11-R	TCTGGAAAGCTAGCACTCAT
