# Supplementary material for: A novel chimeric RNA originating from BmCPV S4 and Bombyx mori HDAC11 transcripts regulates virus proliferation
Source: PLoS Pathog. 2023 Dec 4;19(12):e1011184. doi: 10.1371/journal.ppat.1011184 (PMC10721177; doi:10.1371/journal.ppat.1011184)
Supplement: Attachment — Submitted filename: Response Letter of PPATHOGENS-D-23-00227.docx [file ppat.1011184.s007.docx]

***PLoS Pathogens***

Dear Editors and reviewers,

On behalf of all the contributing authors, I would like to express our sincere appreciation of your kind letter and reviewers’ constructive comments concerning our article entitled "**A novel chimeric RNA originating from BmCPV S4 and Bombyx mori HDAC11 transcripts regulates virus proliferation**" (Manuscript No.: **PPATHOGENS-D-23-00227**). These comments are all valuable and helpful for improving the quality of our article. We are also very grateful to the editor for giving us extra time so that we can carry out new experiments. All the authors have seriously discussed all these comments. According to the reviewers’ objective comments, we have tried our best to modify our manuscript to meet the requirements of your journal and supplemented extra data to make our results convincing.

In the revised version, all modifications are marked in red. Here are our responses to the reviewer's comments point by point. We hope that the quality of the revised manuscript meets the requirements for publication in PLoS *Pathogens*.

If you have any other questions, please don’t hesitate to contact us. We are happy to polish our manuscript to meet your requirements. Thanks again for the opportunity to present our work.

Best Regards.

Chengliang Gong, Jun Pan, Shulin Wei

Corresponding author: Chengliang Gong

School of Biology and Basic Medical Sciences, Soochow University, Suzhou, Jiangsu, 215123, China

Tel.: +86-512-65880183; fax: +86-512-65880183.

E-mail address: gongcl@suda.edu.cn

***********************

Part I – Summary

**Reviewer #1：**

***Pan et al. have discovered several novel chimeric RNAs whereby the silkworm RNA has fused with the BmCPV virus. It has previously been reported that start codons with cap-snatched host transcripts or trans-splicing can generate chimeric RNAs on ssRNA and dsDNA viruses; however, whether chimeric RNAs can be generated on dsRNA viruses remains a mystery. Here the authors use BmCPV (a dsRNA virus) infection of the silkworm to demonstrate the presence of several host-virus chimeras including HDAC11-S4 RNA4, derived from the silkworm histone deacetylase 11 and the BmCPV S4 transcript encoding viral structural protein 4 (VP4).***

***They validated the presence of the RNA chimera using northern blot and in situ hybridization, then showed that the transcript could be translated into a truncated BmCPV CP4 with a silkworm HDAC11-derived N-terminal extension. Further, they found that the chimeric RNA inhibited virus proliferation and decreased histone methylation, and increased histone acetylation.***

**Response:** Thank you very much for summarizing our work so succinctly. It is a great honor to receive your recognition for this work.

**Reviewer #2：**

***The manuscript titled ‘A novel chimeric RNA originating from BmCPV S4 and Bombyx mori HDAC11 transcripts regulates proliferation’ by Pan et al. show the presence of host-viral chimeric RNA in the midgut of BmCPV-infected silkworms. Using RNA_seq and Sanger sequencing they observed the chimera of host histone deacetylase 11 and BmCPV S4 RNA HDAC11-S4 RNA in BmN cells transfected with cDNA of BmCPV S4 both in presence or absence of S2 cDNA encoding RNA dependent RNA polymerase (RdRp), denoting that the chimeric RNA was not mediated by RdRp. The authors make the point that this is the first instance that a RdRp independent chimeric RNA mechanism of synthesis is being observed in silkworm-infected with virus. They further demonstrate the characteristics/fate of the chimeric RNA using qPCR that the expression increases upon infection but decreases 140 hrs after infection, finally showing that these RNAs cannot be packaged into virions. By monitoring the expression of other viral genes like vp1 and protein expression of VP7, they found that the expression of viral genes were decreased when infected with these chimeric RNAs., suggesting that the chimeric RNA causes inhibition of BmCPV replication. They then observe using Western blot that this chimeric HDAC11-S4 RNA decreased trimethylation of histone 3 lysine 9 but increased the histone 3 lysine 9 acetylation, highlighting a possible biological effect of such chimeric RNA on host cells. Western blotting detection using anti-VP4 antibody in the BmCPV infected midgut cells showed not only the presence of viral protein VP4 but also a truncated NesVP4 protein product translated from another ORF starting at the N-terminal extension derived from the host RNA. NesVP4 protein product was also detected when in vitro transcribed HDAC11-S4 mRNA was transfected into the BmN cells.***

***Overall the work is sound on technique and establishes the presence and effect of host-viral chimeric RNA in silkworms upon BmnCPV viral infection. The only issue for this reviewer is the way the text has been presented in the introduction misleads the readers to think that this work is based on yet another host-viral cap-snatching mechanism of chimeric RNA formation. On that note, the text highly resembles that of previously published papers on cap-snatching. While the introduction has tried to summarize all the diverse examples of chimeric host-viral chimeric RNA origins, it fails to lead into the main take-home of this work, which is the identification of a distinct mechanism of chimeric RNA in silkworm-infected with BmnCPV. Although chimeric RNA formation using a cap-snatching mechanism has been previously reported for many different host-virus infections, this work shows and characterizes for the first time the occurrence of silkworm-BmCPV chimeric RNA possibly generated using a different mechanism, further attempting to characterize the biological effect of this chimeric RNA in virus-infected BmN cells.***

**Response:** Thank you very much for your recognition of our researches. We have re-described the contents on the cap-snatching mechanism in the Introduction section in order to avoid misleading readers and to reduce similarity to previously published papers. In addition, the main take-home of this work is introduced in the introduction section in the revised manuscript following the reviewer's suggestions.

**Reviewer #3：**

***Pan et al. identified host-virus chimeric RNAs in a dsRNA virus infection model (BmCPV in silkworm), which is a novel phenomenon. They do follow-up experiments with one specific chimeric RNA and suggest it may be functionally active and biologically relevant.***

***The key experiments do demonstrate that the chimeric protein has activity but fall somewhat short of proving that the chimeric protein provides novel functionality compared to either of the normal versions of the two components on their own.***

**Response:** Thank you for your rigorous comments. We conducted research on the effects of transfection with different doses of chimeric HDAC11-S4 RNA 4 on BmCPV replication and modification of histone H3. Meanwhile, the transfection with the corresponding doses of partial HDAC11 RNA sequence (the upstream sequence of the junction site of chimeric HDAC11 S4 RNA 4), S4 RNA, and GFP RNA were used as controls respectively, following the reviewer’s comments.

The results showed that the level of H3K9me3 decreased, the level of H3K9ac increased, and the protein level of viral VP7 gene and the transcription level of viral *vp1* gene decreased in the transfected BmN cells with the chimeric HDAC11-S4 RNA 4, compared to the transfected BmN cells with partial HDAC11 RNA sequence, viral S4 RNA or GFP RNA (Figure 1), suggesting that the chimeric HDAC11-S4 RNA 4 does provide novel functionality compared to either of the normal versions of the two components on their own. These results have been supplemented into the revised manuscript (Line 258-284), and the Figure 6 in the revised version has been updated.

**Figure 1** Effects of chimeric HDAC11-S4 RNA 4 on BmCPV gene expression and H3K9me3/H3K9ac.

**A**, Schematic diagram of various RNA molecules in the chimeric HDAC11-S4 RNA 4. HDAC11-S4 RNA 4, a chimeric RNA derived from HDAC11 RNA and the BmCPV S4 RNA; partial HDAC11 RNA, the upstream sequence of the junction site of chimeric HDAC11 S4 RNA 4; CPV S4 RNA , The RNA derived from BmCPV segment S4 RNA. **B**, Effect of chimeric HDAC11-S4 RNA 4 on BmCPV VP7 and H3K9me3/H3K9ac of histone 3 levels. BmN cells (10^6^) transfected with 1, 2 or 3 μg of chimeric HDAC11-S4 RNA 4 were inoculated with BmCPV at 24 h posttransfection, and the VP7 protein level and H3K9me3/H3K9ac of histone 3 was determined at 48 h postinfection by Western blotting. Partial HDAC11 RNA, CPV S4 RNA, and GFP RNA(produced by *in vitro* transcription)-transfected cells infected with BmCPV were used as a control. **C-E**, The grayscale intensity of the Western blot signal bands in **Figure 1-B** was analyzed by Image J software. **F-H**, Effect of chimeric HDAC11-S4 RNA 4 on BmCPV *vp1* gene expression levels. BmN cells (10^6^) transfected with 1, 2 or 3 μg of chimeric HDAC11-S4 RNA 4 were inoculated with BmCPV at 24 h posttransfection, and the relative expression level of the BmCPV *vp1* gene was determined at 48 h posttransfection by qRT‒PCR. Partial HDAC11 RNA, CPV S4 RNA, and GFP RNA-transfected cells infected with BmCPV were used as a control. Each experiment was repeated three times. ***, *P*<0.001; **, *P*<0.01; *, *P*<0.05.

Part II – Major Issues

**Reviewer #1：**

***For Figure 4, since the probe targets the junction (i.e., ½ of the probe is binding to the host gene), I am surprised that no signal is observed from the control sample with no infection (I would have expected some binding to this region). Need evidence to show that the probe is binding the junction and not BmCPV on its own.***

***I have the same concern about the FISH probe in figure 4. Need evidence to show that the probe is binding to the junction and not just the BmCPV.***

**Response**: The mature linear RNA molecules is generated from primary transcript through canonical splicing, meanwhile circular RNA (circRNA) may also be formed by back-splicing. The previous reports indicated that the authenticity of circRNAs can be validated by Southern blot and in situ hybridization using an oligonucleotide probe targeting the junction site of circRNA, That is to say, this experiment can distinguish between linear RNA and circular RNA **(Bersani et al., 2023; Zhou et al., 2021).** Therefore, in this study, we used an oligonucleotide probe targeting the junction site of HDAC11-S4 RNA 4 to validate the authenticity of the chimeric RNA through Northern blot and in situ hybridization. Indeed, we understand the reviewer's concerns because half of the probe is binding to the host gene, the other half of the probe is binding to virus S4 RNA. However, the melting temperature (Tm) value of the probe binding to the junction site of the chimeric RNA is significantly higher than the Tm value of the probe binding to the host gene / virus S4 RNA. That is to say, in Northern blot or in site hybridization experiments, by increasing the washing temperature, free probes and probes that do not bind to the junction site of chimeric RNA can be removed. Therefore, in our experiment, no signal is observed from the control sample without infection. To confirm that the probe is binding to the junction site and not to HDAC11 RNA / virus S4 RNA, the prepared HDAC11-S4 RNA 4, HDAC11 RNA, and S4 RNA by *in vitro* transcription were separated by electrophoresis, and then subjected to Northern hybridization using an oligonucleotide probe targeting junction site. Hybridization signal was only detected in HDAC11-S4 RNA 4 sample (Figure 2). Similarly, when *in situ* hybridization was performed on *Ctenopharyngodon idellus* kidney (CIK) cells respectively transfected with HDAC11-S4 RNA 4, partial HDAC11 RNA, and S4 RNA, hybridization signal was only detected in the cells transfected with HDAC11-S4 RNA 4 (Figure 3). These results demonstrated that the hybridization signal comes from the binding of the probe to the junction site, rather than the host HDAC11 RNA or the viral S4 RNA. These results have been supplemented in the revised manuscript as Figure S3. (Line 219-229)

**Figure 2** Northern blotting for HDAC11-S4 RNA 4, partial HDAC11 RNA, and CPV S4 RNA. HDAC11-S4 RNA 4, partial HDAC11 RNA, and CPV S4 RNA prepared by *in vitro* transcription were separated on 1% agarose-formaldehyde gels and transferred to Hybond-N+ nylon membranes. Northern blotting was conducted with biotin-labeled DNA targeting the junction site of chimeric HDAC11-S4 RNA 4.

**Figure 3** *In situ* hybridization of *Ctenopharyngodon idellus* kidney cells respectively transfected with HDAC11-S4 RNA 4, partial HDAC11 RNA, and CPV S4 RNA.

CIK cells (1×10^4^) were seeded in 24-well plates, and followed by transfection with partial HDAC11 RNA (2μg), CPV S4 RNA (2μg) or HDAC11-S4 RNA 4 (2μg). The cells collected at 48 h post-transfection were subjected to in situ hybridization with biotin-labeled DNA targeting the junction site of chimeric HDAC11-S4 RNA 4. Hybridization signals were detected using CY3-labeled streptavidin. Cell nuclei were counterstained with DAPI.

Bersani F, Picca F, Morena D, Righi L, Napoli F, Russo M, Oddo D, Rospo G, Negrino C, Castella B, Volante M, Listì A, Zambelli V, Benso F, Tabbò F, Bironzo P, Monteleone E, Poli V, Pietrantonio F, Di Nicolantonio F, Bardelli A, Ponzetto C, Novello S, Scagliotti GV, Taulli R. Exploring circular MET RNA as a potential biomarker in tumors exhibiting high MET activity. J Exp Clin Cancer Res. 2023, 42(1):120.

Zhou J, Wang L, Sun Q, Chen R, Zhang C, Yang P, Tan Y, Peng C, Wang T, Jin C, Ji J, Jin K, Sun Y. Hsa_circ_0001666 suppresses the progression of colorectal cancer through the miR-576-5p/PCDH10 axis. Clin Transl Med. 2021,11(11):e565.

**Reviewer #2：**

**Response**: Thank you very much. It is a great honor to receive your recognition for this work!

**Reviewer #3：**

***1. Lines 222-224: No data are shown to support the claim that the chimeric HDAC11-S4 RNA 4 is not packaged into virions.***

**Response**: We detected RNA in virus particles through RT-PCR, and the RT-PCR products were separated by electrophoresis. No specific band representing chimeric HDAC11-S4 RNA 4 was observed, therefore, we did not include this electrophoretogram in our manuscript. In the revised version, we have added a description of "data not shown" in the relevant results. ( Line 251-252 )

***2. Figure 6 and related text:***

***o What was the rationale for choosing chimeric HDAC11-S4 RNA 4 for further workup? Was it the most abundant? Or was it chosen randomly?***

**Response**: Thank you for your question. In this study, 6 isoforms (HDAC11-S4 RNA 1-6) of HDAC 11-S4 RNA were identified, in which, HDAC11-S4 RNA 4 may have the potential to encode a truncated viral VP4 with N-terminal extensions derived from host HDAC11 RNA, therefore, we chose HDAC11-S4 RNA 4 for further research. We have described the rationale in the revised manuscript. (Line 255-258)

***3. Figure 6 and related text:***

***o Why did the authors not choose wild-type HDAC11 and/or the short N-terminal fragment found in the chimeric protein and/or wild-type VP4 as a control for this experiment, and also transfect that in increasing doses? This would not only be a better control in general (see my next point), it would also let them address whether this chimeric protein offers any additional functionality compared to the normal cellular HDAC11 and the normal viral VP4.***

**Response:** We are all very recognition of your useful pertinent proposal. Following the reviewer’s comments, the effect of transfection with different doses of chimeric HDAC11-S4 RNA 4 on BmCPV replication and modification of histone H3 were investigated. Meanwhile, the transfection with the corresponding doses of CPV S4 RNA, partial HDAC11 RNA (upstream sequence of the junction site of chimeric HDAC11 S4 RNA 4), and GFP RNA were used as controls respectively. The results were presented in Figure 1.

***o To demonstrate that the changes in vp1 RNA, VP7 protein, H3K9me3 and H3K9ac are all specific effects mediated by increasing dosages of HDAC11-S4 RNA 4, all experiments shown in this figure should be repeated with matching increasing dosages of a control RNA. Using only a single dose of GFP RNA doesn’t rule out non-specific effects.***

**Response**: We are all very recognition of your useful pertinent proposal. Following the reviewer’s comments, the effect of transfection with different doses of chimeric HDAC11-S4 RNA 4 on BmCPV replication and modification of histone H3 were investigated. Meanwhile, the transfection with the corresponding doses of partial HDAC11 RNA, CPV S4 RNA, and GFP RNA were used as controls respectively. The results showed that the *vp1* gene transcription level and VP7 protein level decreased, the H3K9me3 level decreased and the H3K9ac level increased in the chimeric HDAC11-S4 RNA 4-transfected BmN cells in a transfection dose-dependent manner, compared to GFP RNA-transfected BmN cells (Figure 1). Compared to partial HDAC11 RNA- and CPV S4 RNA-transfected BmN cells, the VP7 protein level and the *vp1* transcription level decreased, and the H3K9me3 level decreased and the H3K9ac level increased in the transfected BmN cells with HDAC11-S4 chimeric RNA 4, suggesting that the chimeric HDAC11-S4 RNA 4 provides novel functionality compared to either of the normal versions of the two components on their own. These results were added in the revised manuscript. (Line 258-284) Meanwhile, Figure 6 has also been updated accordingly.

Part III – Minor Issues

**Reviewer #1：**

1. ***Sometimes RT-PCR is referred to as PCR in the text***

**Response**: Thank you. We have carefully checked the full text and made corresponding corrections.

2. ***It would be helpful if the sequences for BMSK_chr11 were aligned in Figure 2a and 2b (they are currently offset by a few nucleotides) and a reference sequence for BMSK_chr11 the viral inserts were aligned there as well.***

**Response**: We have revised it following reviewer’s comments.

3. ***Same comment above for the other examples in figure 2 and figure 3, reference sequences would be helpful to see sequence similarities, etc.***

**Response**: Thanks for your advice. We have remade Figure 3 following the reviewer's comments.

4. ***Did all instances of the host transcripts found in chimera form become this way, or is there still a fraction of these transcripts that does not become a chimera? Can the authors comment on or estimate the percentages?***

**Response**: Thank you for your question. We estimated the ratio of HDAC11-S4 RNA 4, S4 RNA, and HDAC11 RNA in BmCPV-infected BmN cells at 48 h post-infection using RT-qPCR, and the results showed that HDAC11-S4 RNA 4: S4 RNA: HDAC11 RNA was 1:19:139, indicating that only a fraction of CPV S4 RNA and HDAC11 RNA become chimeric RNAs. What’s more, this result has been described in the revised version. (Line 247-250)

5. ***For figure 4, the full gel, including the size markers, should be shown rather than a cutout.***

**Response**: We re-conducted the Northern blot experiment. The results were presented in Figure 4, and added to the revised manuscript following reviewer’s requirement.

**Figure 4** Validation of HDAC11-S4 RNA 4 by Northern blotting.

The total RNA extracted from the BmCPV-infected midgut was separated on 1% agarose-formaldehyde gels and transferred to Hybond-N+ nylon membranes. Northern blotting was conducted with biotin-labeled DNA targeting the junction site of chimeric HDAC11-S4 RNA 4. Total RNA extracted from the non-BmCPV-infected midgut was used as a control. Lane M, DNA marker; lane Con, the non-BmCPV-infected midgut RNAs；lane BmCPV, BmCPV-infected midgut RNAs.

6. ***In the methods section where the FISH experiment is described, there is a typo–streptomycin should be cy3-labeled streptavidin, and the images in figure 4 should have scale bars.***

**Response:** Thank you very much. The typo-streptomycin has been corrected and the scale bars have been added to the images in Figure 4 following reviewer’s comments.

7. ***Axis labels for figure 5***

**Response:** The axis labels for Figure 5 have been added in the revised manuscript according to the comments.

**Reviewer #2：**

***Line 268 - Rephrase for grammar error, maybe like - ‘because the host genomic DNA region integrates viral DNA and then is transcribed’***

**Response:** Thank you very much for your careful reading and pointing out the grammar error, we have made correction as you pointed out.

**Reviewer #3：**

1. ***• Figure 6 and related text:***

***o If the authors want to suggest that the chimeric protein encoded by HDAC11-S4 RNA 4 is potentially biologically relevant, they should comment on its abundance. If the RNA is vanishingly rare, it will likely not matter in vivo. Their NGS data should enable the authors to discuss this point.***

**Response:** Thank you very much for your constructive comments. The junction read count obtained by high-throughput sequencing (Table S1) suggested that the abundance of HDAC 11-S4 RNA was not high. In this study, 6 isoforms of HDAC 11-S4 RNA 4 were identified by RT-PCR and Sanger sequencing. Our qRT-PCR results showed that HDAC11-S4 RNA 4 : CPV S4 RNA : HDAC11 RNA was 1:19:139. Moreover, CPV S4 RNA encodes the viral structural protein VP4, and the abundance of S4 RNA increases with virus proliferation. Besides, Western blot results showed that the level of NesVP4 protein encoded by the chimeric HDAC11-S4 RNA 4 was comparable to the level of VP4 protein (Figure 7), suggesting that the NesVP4 chimeric protein encoded by HDAC11-S4 RNA 4 is potentially biologically relevant. We have commented on its abundance in the revised manuscript. (Line247-250)

2. ***Lines 189-192: RNA 4 also does not match the reference sequence (see fig.3D). It has a 1nt mismatch (T895A). Do the authors think this is an artifact? Would this affect the amino acid sequence?***

**Response:** Thank you very much for your careful and seriousness. We used RT-PCR and Sanger sequencing to determine the flanking sequence of the junction site of HDAC11-S4 RNA 4. The results showed that the base at position 895 of S4 RNA is ‘T’, but there is also case where it is A (Figure 6). We think this result is related to the mutation of some virus S4 RNA sequences, rather than artifact. T895A mutation does not affect the amino acid sequence, because this point mutation occurs at the third base of the codon. We updated Figure 3D and explained in the revised manuscript. (Line 203-207 )

**Figure 5** The flanking sequence of the junction site of HDAC11-S4 RNA 4

3. ***Figure legends are missing important information, for example:***

***o Figure 1A: What do the red and blue lines in the centers of the Circos plots indicate?***

**Response:** Each line in the Circos plots represents a fusion event; the red line represents the fusion event between two RNA molecules originating from the same chromosome, while the blue line represents the fusion event between RNA molecules originating from different chromosomes. It has been indicated in the revised version. (Line 696-700)

***o Figure 1A: The legend should say that“BMSK_x” indicates the individual silkworm chromosomes.***

*Response：*We have done following reviewer’s comments. (Line 695-696)

***o Figure 1B: What are the circles at one end of the wavy lines representing transcripts?***

**Response:** The circle at one end of the wavy line represents the 5’-end of RNA molecule. We have described in the revised manuscript.

***o Figures 5 and 6: What is the basis for the error bars? How many replicates are shown? Biological replicates or technical replicates? Is any of it significant?***

**Response:** The error bar represents the error between three repeated biological experiments. Statistical analysis of the experimental results was conducted, and the differences' significance has been indicated in the figure. It has been explained in the updated Figure legends.
